# Multicenter clinicopathological study of odontogenic myxoma spectrum lesions using quantitative pathology

**DOI:** 10.1038/s41598-026-42019-8

**Published:** 2026-02-27

**Authors:** Yosuke Harazono, Yuki Fukawa, Takuya Iwasaki, Yohei Hama, Naoto Nishii, Yosuke Tanaka, Hikari Saito, Hideaki Kamochi

**Affiliations:** 1 Department of Maxillofacial Surgery, Graduate School of Medical and Dental Sciences, Institute of Science Tokyo, 1-5-45 Yushima, Bunkyo-ku, Tokyo, 113-8510 Japan; 2 Department of Oral Pathology, Graduate School of Medical and Dental Sciences, Institute of Science Tokyo, 1-5-45 Yushima, Bunkyo-ku, Tokyo, 113-8510 Japan; 3 Department of Gerodontology and Oral Rehabilitation, Graduate School of Medical and Dental Sciences, Institute of Science Tokyo, 1-5-45 Yushima, Bunkyo- ku, Tokyo, 113-8510 Japan; 4 Department of Oral and Maxillofacial Surgical Oncology, Graduate School of Medical and Dental Sciences, Institute of Science Tokyo, 1-5-45 Yushima, Bunkyo-ku, Tokyo, 113-8510 Japan; 5 Department of Human Genetics and Disease Diversity, Graduate School of Medical and Dental Sciences, Institute of Science Tokyo, 1-5-45 Yushima, Bunkyo-ku, Tokyo, 113-8510 Japan; 6 Division of Surgical Pathology, Institute of Science Tokyo hospital, 1-5-45 Yushima, Bunkyo-ku, Tokyo, 113-8510 Japan

**Keywords:** Odontogenic myxoma, Odontogenic myxofibroma, Quantitative pathology, Clinicopathological correlation, Digital pathology, Radiological morphology, Cancer, Diseases, Medical research, Oncology

## Abstract

Odontogenic myxoma (OM) and odontogenic myxofibroma (OMF) are rare benign odontogenic tumors characterized by heterogeneous stromal composition, for which objective and reproducible pathological evaluation remains challenging. This multicenter study aimed to quantitatively assess fibrous tissue proportion (FTP) using AI-assisted digital pathology and to explore its clinicopathological relevance across odontogenic myxoma spectrum lesions. A total of 143 surgical specimens were collected from 34 institutions, and 100 cases were included after centralized pathological review. FTP was independently estimated by two board-certified oral pathologists to generate expert reference data. Whole-slide images of Masson’s trichrome–stained sections were analyzed using a multi-stage deep learning pipeline implemented within a unified digital pathology platform. Agreement between expert assessment and quantitative measurements was evaluated, and associations between FTP and clinical variables were explored. Quantitative FTP measurements showed good agreement with expert pathological evaluation and revealed substantial inter-institutional variability in pathological diagnoses. In clinicopathological analyses, higher FTP was independently associated with unilocular radiological morphology. These findings demonstrate that AI-assisted quantitative pathology provides a reproducible framework for visualizing stromal heterogeneity in odontogenic myxoma spectrum lesions and may support more consistent clinicopathological interpretation across institutions.

## Introduction

Odontogenic myxoma (OM) is a benign tumor characterized by collagen fibers within a myxoid stroma^1,2^. It accounts for approximately 2–5% of all odontogenic tumors, with an estimated incidence of 0.07 per million population^3–7^. In contrast, odontogenic myxofibroma (OMF) is a benign tumor containing a greater proportion of fibrous tissue than OM. OMF has been suggested to result from myxomatous degeneration of odontogenic fibroma^8,9^. The clinical differences between these lesions and the clinical significance of their classification within the odontogenic myxoma spectrum lesions remain unclear, resulting in the absence of established diagnostic criteria. Consequently, differential diagnosis currently depends on the judgment of pathologists at individual institutions.

In our previous study, we compared OM and OMF, focusing on fibrous tissue proportion (FTP), and demonstrated a significant correlation between FTP and the final pathological diagnosis^10^. However, although trends were noted in cortical bone perforation and magnetic resonance imaging (MRI) apparent diffusion coefficient (ADC) values, a clear correlation between FTP and clinical findings was not established. To the best of our knowledge, this report of 21 cases constitutes the largest consecutive single-institution case series of OM and OMF, excluding review articles^11–14^. Accordingly, larger studies employing robust, reproducible analyses under standardized conditions are required to elucidate their pathophysiology further.

This study aimed to evaluate stromal heterogeneity within odontogenic myxoma spectrum lesions by expanding the case series and applying image analysis software to quantify FTP. The present work represents the largest multicenter collaborative analysis of OM and OMF conducted under standardized conditions.

## Materials and methods

### Samples

Patients diagnosed with OM or OMF who underwent surgical treatment at 34 oral and maxillofacial surgery departments between 2004 and 2024 were included, yielding a total of 143 specimens. Data extracted from medical records included patient age and sex; location (maxilla or mandible); duration of illness; presence of pain, swelling, and nerve paralysis; lesion size (maximum diameter on computed tomography images [CT] images); presence of cortical bone perforation; locularity (unilocular or multilocular); associated teeth root resorption or displacement; ADC values from MRI; surgical procedure (dredging method [D], enucleation [E], enucleation + curettage [EC], enucleation + peripheral osteotomy [EP], marginal resection [M], segmental resection [S]); final clinicopathological diagnosis; follow-up period; and recurrence. Three cases diagnosed only by biopsy, without subsequent surgical resection, were excluded.

This retrospective observational study was conducted in accordance with the ethical principles of the Declaration of Helsinki and the Ethical Guidelines for Medical and Health Research Involving Human Subjects established by the Ministry of Health, Labour and Welfare of Japan. Ethical approval was obtained from the Institutional Review Board of the Institute of Science Tokyo (D2024-014). The requirement for informed consent was waived owing to the retrospective design of the study.

### Histopathological analysis and quantitative image analysis

**Fig. 1:**
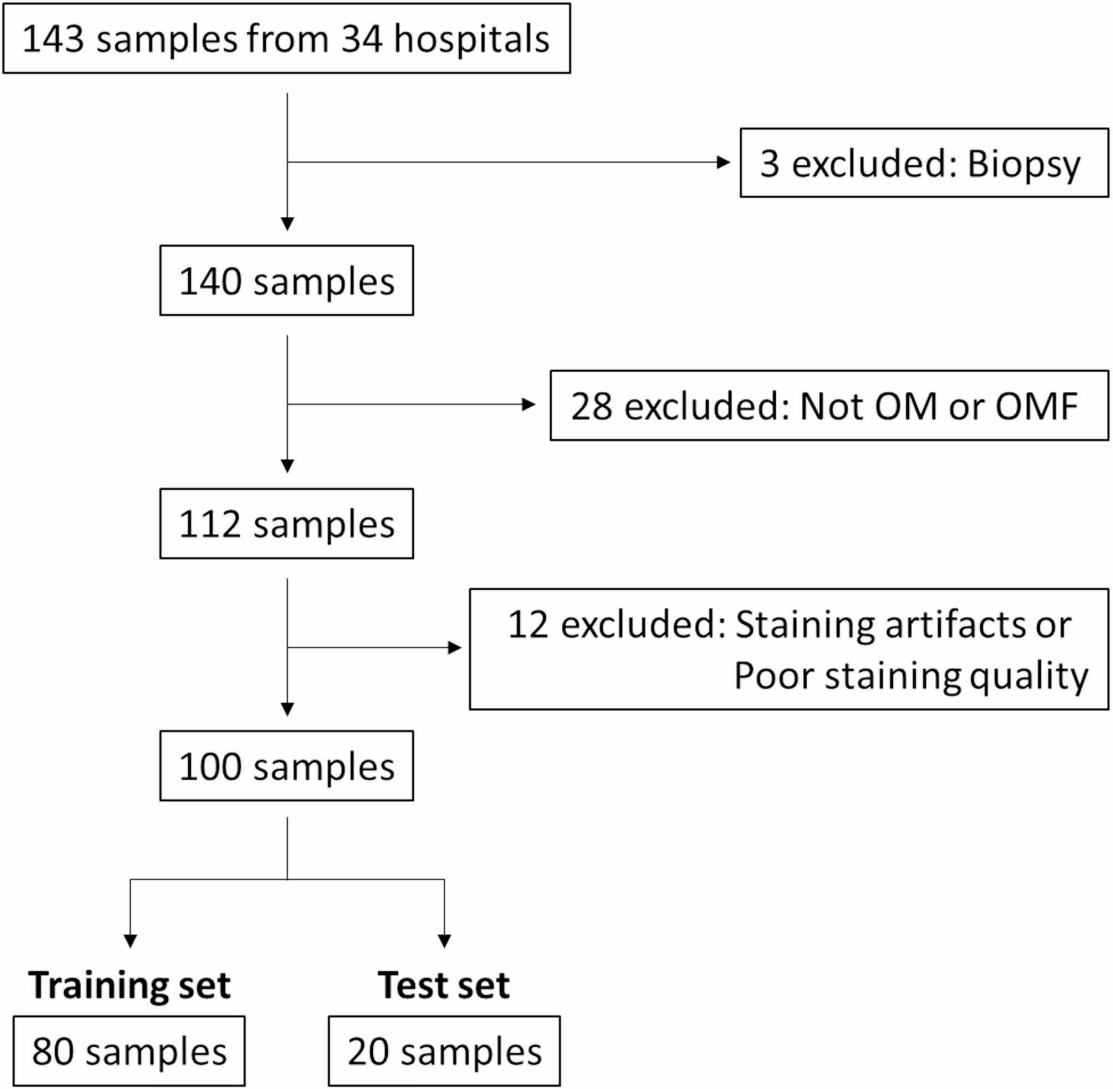
Case selection flow diagram for the multicenter study.

To ensure diagnostic consistency, all cases diagnosed as OM or OMF were subjected to central review by a board-certified oral pathologist (Y.F.) according to the current WHO classification. Subsequently, cases were excluded based on the following criteria: a diagnosis other than OM or OMF; insufficient specimen size for fibrous tissue assessment; or extensive secondary inflammation hindering the evaluation of fibrous tissue. For fibrous tissue quantification, representative formalin-fixed paraffin-embedded blocks were sectioned at 4 μm thickness and stained with Masson’s trichrome (MT) under standardized conditions using an automated system (Tissue-Tek Prisma, Sakura Finetek Japan Co., Ltd., Tokyo, Japan). A total of 100 cases were included in the final analysis, with 80 randomly assigned to the training algorithms and 20 to the test algorithms (Fig. 1). All cases were independently evaluated by two board-certified pathologists (Y.F and Y.T.), who estimated the fibrous tissue proportion within the tumor in 10% increments to generate the expert reference data; these values were defined as the expert-assessed fibrous tissue proportion (Expert FTP). Interobserver agreement was assessed using the intraclass correlation coefficient (ICC), calculated with a two-way random-effects model for single measurements (ICC (2,1)). Discrepancies between the two assessments were reviewed during central pathological evaluation, and final reference values were determined by consensus.

A total of 143 samples were collected from 34 institutions. Among these, 3 biopsy-only cases, 28 cases diagnosed as entities other than OM or OMF, and 12 cases with staining artifacts or inadequate staining quality were excluded. The remaining 100 samples were included in the final analysis, with 80 assigned to the training set and 20 to the test set for AI model development.

**Fig. 2:**
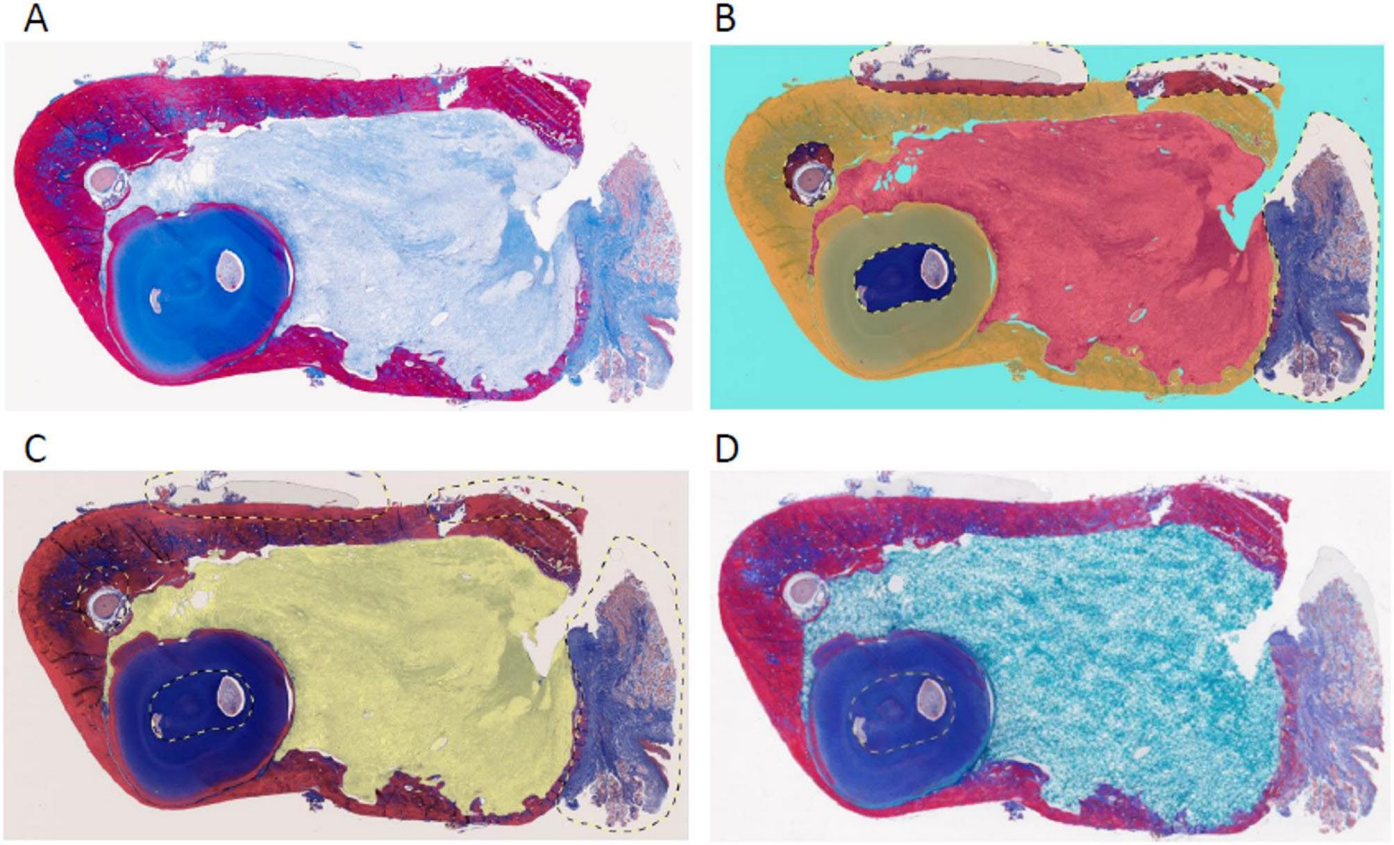
Hierarchical AI training and analysis pipeline for fibrous tissue quantification in HALO. (**A**) Masson’s trichrome (MT)–stained whole-slide image. (**B**) Tissue region segmentation, in which the HALO AI algorithm distinguishes tissue from background. (**C**) Tumor region extraction, with classification of tumor tissue and exclusion of non-tumor elements, including bone, tooth structures, and gingival tissue. (**D**) Non-nuclear region extraction and subsequent identification of MT-positive fibrous tissue, followed by calculation of the fibrous tissue proportion (FTP) as the area of fibrous tissue relative to the tumor area.

Whole-slide images (WSIs) of all MT-stained sections were acquired using a NanoZoomer S210 digital slide scanner (Hamamatsu Photonics K.K., Hamamatsu, Japan) at 20× magnification, corresponding to a resolution of 0.46 μm/pixel. WSIs were imported into the image analysis software HALO version 4.1 (indica Labs, NM, US) for annotation, training, and classification. A commercially available and widely used digital pathology platform was selected to prioritise reproducibility, transparency, and transferability of the analytical workflow across institutions. Tissue classification was performed using the HALO Tissue Classifier analysis module (random forest algorithm) and HALO AI (convolutional neural network [CNN], VGG architecture). The proposed analysis pipeline consisted of three sequential deep learning classes: (1) tissue region segmentation, (2) tumor region extraction, and (3) non-nuclear region extraction and subsequent fibrous component quantification. Each class was trained independently and integrated into a single inference pipeline. The first class segmented tissue from background, the second distinguished tumor from non-tumor regions, and the third separated non-nuclear from nuclear areas and identified fibrous tissue. Annotations for training the tissue classification algorithms were provided by a board-certified oral pathologist (Y.F.), with collaborative assistance from a colleague (Y.H.). Approximately 4,000 annotations, including various polygonal outlines, were provided for training each class. Training and classification were performed at high resolution for 15,000 iterations. In the third stage, nuclear regions were excluded using the Indica pretrained BF Nuclei Seg network, and fibrous components were subsequently quantified within the remaining non-nuclear areas. Ultimately, FTP was calculated as the area of “fibrous tissue” relative to the “tumor” area (Fig. 2); this value was defined as the AI-estimated fibrous tissue proportion (AI FTP).

The final AI model was optimized in the training set by fitting its outputs to the expert annotations of two board-certified pathologists using the least-squares method, and its performance was subsequently validated in the test set. The association between Expert FTP and AI FTP was assessed using Spearman’s rank correlation coefficient. Agreement between the two measurements was evaluated using the ICC and Bland–Altman–type plots, with Expert FTP serving as the reference standard. The AI model was designed as a quantitative measurement tool for stromal composition rather than as a diagnostic classifier, thereby minimizing sensitivity to inter-institutional diagnostic bias and enhancing reproducibility in multicenter settings.

### Statistical analyses

For each clinical variable, associations with AI FTP were examined, with AI FTP treated as the dependent variable. Data normality was assessed using the Shapiro–Wilk test. For comparisons between two groups defined by binary categorical variables, Student’s t-test was applied when normality was satisfied and homoscedasticity was confirmed by Levene’s test; Welch’s t-test was used when homoscedasticity was violated. When the assumption of normality was not met, the Wilcoxon rank-sum test was performed. Associations between AI FTP and continuous variables were evaluated using Pearson’s correlation coefficient or Spearman’s rank correlation coefficient, as appropriate based on data distribution.

Multivariable analysis was conducted on an exploratory basis, with variables selected based on a combination of potential associations observed in the univariate analyses and clinical relevance, rather than strict statistical thresholds, to avoid excluding potentially meaningful factors in this rare-disease cohort. A multiple linear regression analysis using the forced-entry method was performed with AI FTP as the dependent variable. The significance level was set at 0.05. All statistical analyses were conducted using JMP Student Edition version 19.0.1 (SAS Institute Inc., Cary, NC, USA). Given the rarity of odontogenic myxoma spectrum lesions, the multivariable analysis was conducted to explore potential clinicopathological associations rather than to develop a predictive model. Accordingly, variable selection prioritized clinical interpretability and biological plausibility in addition to trends observed in univariate analyses.

## Results

### Sample demographics

Table 1 presents the demographic data of the 100 included cases, based on the clinical and pathological diagnoses made at each participating institution. No clinical features differed significantly between OM and OMF as diagnosed at these institutions.

**Table 1:** Clinical features of OM and OMF.

Clinical factor	Clinical and pathological diagnosis		
	OM (*n* = 68)		OMF (*n* = 32)
Age (years, mean ± SD)	39.78 ± 13.85		38.69 ± 16.34
Sex (Male/Female)	31/37		14/18
Location (Maxilla/Mandible)	26/42		7/25
Duration of illness (months, median [IQR])	5 (2–19.5)		6 (1.75–21.5)
Pain (−/+/ND)	53/13/2		22/8/2
Swelling (−/+/ND)	17/49/2		12/19/1
Nerve paralysis (−/+/ND)	59/4/5		26/3/3
Lesion size (mm, mean ± SD)	39.26 ± 19.38		38.91 ± 19.03
Cortical bone perforation (−/+/ND)	19/46/3		13/19/0
Locularity (Uni/Multi/ND)	23/44/1		14/18/0
Teeth resorption (−/+/ND)	54/12/2		29/3/0
Teeth displacement (−/+/ND)	47/19/2		25/7/0
ADC value (mean ± SD)	2.01 ± 0.29		2.07 ± 0.36
Surgical procedure (D/E/EC/EP/M/S/ND)	1/2/10/4/22/28/1		2/2/7/6/7/8/0
Follow-up period (months, median [IQR])	66 (34.5–105.8)		48 (23–85)
Recurrence (−/+/ND)	63/2/3		31/0/1

*OM* odontogenic myxoma, *OMF* odontogenic myxofibroma, *SD* standard deviation, *IQR* interquartile range, *ND* not described, *ADC* apparent diffusion coefficient, *D* dredging method, *E* enucleation, *EC* enucleation + curettage, *EP* enucleation + peripheral osteotomy, *M* marginal resection, *S* segmental resection.

### Model development

**Fig. 3:**
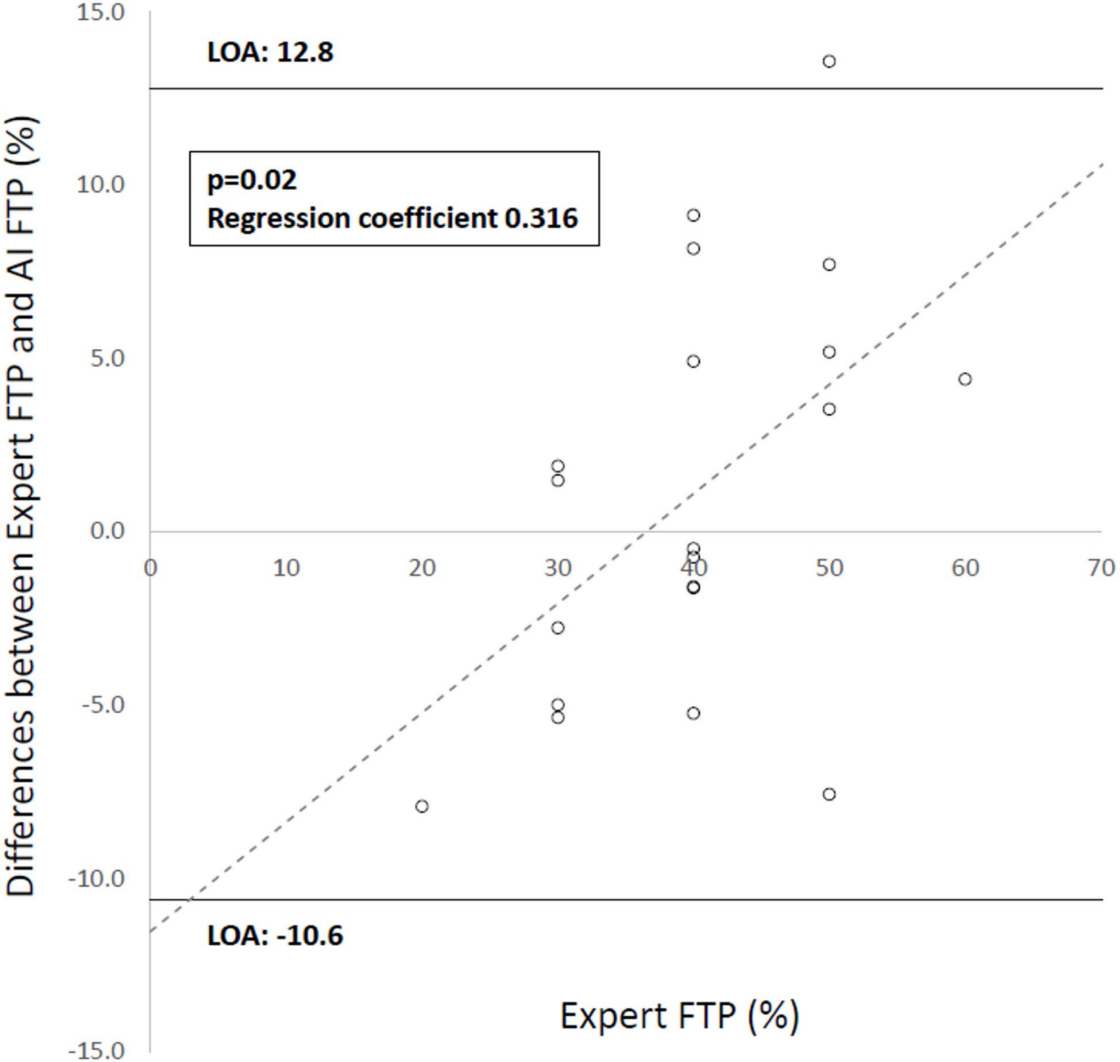
Bland–Altman analysis comparing Expert FTP and AI FTP. The differences between Expert FTP and AI FTP (Expert FTP − AI FTP) are plotted against Expert FTP. Solid lines indicate the 95% limits of agreement (− 10.6 to 12.8), and the dotted line represents the regression line demonstrating a significant proportional bias (regression coefficient = 0.316, *p* = 0.02). Expert FTP, expert-assessed fibrous tissue proportion; AI FTP, AI-estimated fibrous tissue proportion.

Interobserver agreement for FTP assessment between the two expert pathologists was good, with an ICC (2,1) of 0.8586 (95% CI, 0.804–0.907; *n* = 100). The AI model was finalized at the point at which the mean squared difference from the Expert FTP was minimized in the training set (mean ± SD: 1.40 ± 1.92). Its performance was further evaluated in the test set, in which the mean squared difference was 0.88 ± 1.04. Spearman’s rank correlation analysis demonstrated a strong positive correlation between the Expert FTP and AI FTP (ρ = 0.8038). In addition, the ICC (3,1) indicated good agreement between the two measurements (ICC = 0.7853). The mean difference between the Expert FTP and AI FTP was small, and 95% of the differences were within the limits of agreement (− 10.6 to 12.8), indicating a potential deviation of approximately ± 10 at the individual case level. A Bland–Altman–type analysis revealed a significant positive proportional bias between the differences (Expert FTP − AI FTP) and the Expert FTP values (regression coefficient = 0.316, *p* = 0.02), with greater disagreement observed at higher FTP values (Fig. 3). Based on these results, no evidence of overfitting was observed, and the AI model was considered to demonstrate good generalizability, maintaining strong agreement with expert assessments in independent cases. Accordingly, the model was applied to subsequent analyses.

### Association between fibrous tissue proportion and clinical features

Associations between the AI FTP and clinical variables were examined. The results of two-group comparisons and correlation analyses are presented in Table 2. As an exploratory analysis, variables showing potential associations in the univariate analyses, together with clinical relevance, were considered for inclusion in a multivariable model. Accordingly, locularity, swelling, and cortical bone perforation—variables showing potential associations in the univariate analyses and considered clinically relevant—were entered into a forced-entry multiple regression model with AI FTP as the dependent variable. The overall multiple regression model demonstrated marginal statistical significance (*p* = 0.069). Within this model, locularity was independently associated with AI FTP, with unilocular lesions exhibiting a significantly higher proportion of fibrous tissue (partial regression coefficient = 3.69, *p* = 0.0237). In contrast, swelling and cortical bone perforation were not significantly associated with AI FTP. No evidence of multicollinearity was observed, with variance inflation factor (VIF) values ranging from 1.12 to 1.33. (Table 3).

**Table 2:** Univariate analysis of AI FTP according to clinical factors.

Clinical factor	Group 1	Group 2	Test	*p*-value
Age	–	–	Spearman’s ρ = −0.047	0.64
Sex (Male/Female)	40.70 ± 2.17	39.98 ± 1.98	Student’s t-test	0.81
Location (Maxilla/Mandible)	40.64 (31.00–45.03)	37.44 (30.84–51.18)	Wilcoxon rank-sum test	0.65
Pain (−/+)	40.82 ± 1.70	39.32 ± 3.20	Student’s t-test	0.68
Swelling (−/+)	38.45 ± 2.70	41.29 ± 1.77	Student’s t-test	0.38
Nerve paralysis (−/+)	40.67 ± 1.60	42.41 ± 5.57	Student’s t-test	0.76
Lesion size	–	–	Spearman’s ρ = 0.080	0.43
Cortical bone perforation (−/+)	41.86 ± 2.56	39.26 ± 1.80	Student’s t-test	0.41
Locularity (Uni/Multi)	40.49 (32.30–54.92)	36.94 (28.98–44.93)	Wilcoxon rank-sum test	0.098
Teeth resorption (−/+)	37.95 (30.84–50.48)	35.22 (30.87–58.24)	Wilcoxon rank-sum test	0.86
Teeth displacement (−/+)	39.16 (30.34–51.32)	35.79 (31.07–46.66)	Wilcoxon rank-sum test	0.90
ADC	-	-	Spearman’s ρ = −0.004	0.98

Data are presented as mean ± standard deviation or median (interquartile range), as appropriate.

*AI FTP* AI-estimated fibrous tissue proportion, *SD* standard deviation, *IQR* interquartile range, *ADC* apparent diffusion coefficient.

**Table 3:** Multiple regression analysis of AI FTP.

Variable	*p*-value	95% confidence interval	B	VIF
Locularity (Uni)	0.024*	0.51–6.88	3.69	1.12
Swelling (+)	0.101	−0.61–6.77	3.08	1.33
Cortical bone perforation (−)	0.382	−1.96–5.08	1.56	1.30

*AI FTP* AI-estimated fibrous tissue proportion, *B* partial regression coefficient, *VIF* variance inflation factor.

*p < 0.05.

Adjusted R^2^ = 0.04.

## Discussion

The present multicenter study demonstrates that stromal composition in odontogenic myxoma spectrum lesions can be quantitatively and reproducibly assessed using AI-assisted digital pathology under standardized conditions. Because OM is rare, pathological diagnosis has traditionally relied on subjective visual estimation within individual institutions, making systematic evaluation of inter-institutional variability difficult. By applying a unified quantitative framework to specimens collected from 34 institutions, the present study enables objective visualization of stromal heterogeneity across routine clinical settings.

OM and OMF have historically been described based on relative proportions of myxoid and fibrous stroma^15,16^. Although they are currently regarded as synonymous entities in the World Health Organization classification, variability in pathological interpretation and reported clinicopathological features persists^3,17,18^. The absence of standardized quantitative criteria for stromal assessment has likely contributed to this inconsistency, underscoring the need for objective and reproducible evaluation methods^17–24^.

Using a three-stage deep learning pipeline applied to whole-slide images, implemented within an established digital pathology and AI-assisted image analysis platform^25–28^, we quantified FTP and demonstrated strong agreement with expert assessment. Importantly, this approach is intended as a measurement tool for stromal composition rather than as a diagnostic classifier, thereby minimizing sensitivity to institutional diagnostic bias. The multicenter design allowed direct assessment of diagnostic variability under uniform pathological conditions.

Importantly, this study does not aim to redefine OM and OMF as distinct disease entities. Instead, our objective is to establish a reproducible methodological framework for quantifying stromal heterogeneity within the odontogenic myxoma spectrum lesions and to examine how such heterogeneity relates to clinicoradiological features and current diagnostic practice, particularly in the context of minimizing institutional bias in multicenter settings. To illustrate the extent of diagnostic variability under standardized conditions, we applied a provisional FTP cutoff value of 33% as a pragmatic reference point. This cutoff was not intended as a definitive diagnostic threshold or as a proposal for disease reclassification, but solely as an analytical tool to visualize discrepancies between institutional diagnoses and centralized quantitative assessment. The FTP cutoff was applied solely for exploratory illustration and not as a definitive biological or clinical threshold; because all primary analyses treated FTP as a continuous variable, the main conclusions do not depend on the specific cutoff selected, and the interpretation of diagnostic discordance would be unlikely to change materially with alternative illustrative thresholds. The observed discordance underscores the extent to which subjective interpretation of stromal composition may influence pathological diagnosis in routine practice.

Given the rarity of odontogenic myxoma spectrum lesions and the complexity of stromal architecture, the multivariable analysis was conducted in an exploratory manner. Variable selection was guided by a combination of univariate trends and clinical plausibility rather than strict statistical thresholds. Although the adjusted R² value was low, indicating that routine clinical variables explain only a limited proportion of variance in stromal composition, the overall model demonstrated marginal statistical significance, and locularity remained independently associated with AI FTP. Specifically, unilocular lesions exhibited a higher AI FTP, a finding that is biologically plausible given that multilocular lesions typically contain more fluid-rich components and less dense fibrous stroma. Rather than implying predictive utility, these results support the construct validity of quantitative FTP by demonstrating meaningful associations with radiological morphology.

Several limitations warrant consideration. Despite being the largest multicenter cohort analyzed under standardized conditions, the sample size remains constrained by disease rarity. FTP was quantified using representative histological sections rather than whole-tumor sampling. As a result, spatial intratumoral heterogeneity, particularly gradual transitions between myxoid and fibrous components, may not be fully captured. The observed proportional bias at higher FTP values may reflect technical factors, such as limited discrimination between densely collagenous and adjacent stromal components in Masson’s trichrome–stained sections, as well as biological factors related to increased structural complexity in tumors with abundant fibrous stroma. This effect is unlikely to materially affect the interpretation of clinicopathological associations across the full spectrum of stromal composition. Expert assessment served as the reference standard, and interobserver variability is unavoidable. In addition, clinical outcomes such as recurrence could not be robustly evaluated due to low event rates and heterogeneous surgical management. Larger cohorts, whole-tumor sampling, and longitudinal outcome data will be required to further validate this framework.

## Conclusions

In summary, this multicenter study demonstrates that AI-based quantitative pathology provides a reproducible framework for assessing stromal heterogeneity within the odontogenic myxoma spectrum lesions under standardized conditions. By quantitatively visualizing differences in FTP, this approach highlights substantial inter-institutional diagnostic variability and reveals biologically plausible associations with radiological morphology. Rather than redefining disease boundaries, the present framework supports objective comparison of pathological assessments across institutions and may contribute to more consistent clinicopathological interpretation of rare odontogenic tumors.

## Data Availability

The data that support the findings of this study are not publicly available due to ethical and privacy restrictions, but are available from the corresponding author upon reasonable request.

## Declarations

### Generative AI and AI-assisted technologies

Generative AI and AI-assisted technologies (e.g., ChatGPT) were used to improve language clarity during manuscript preparation. All content was reviewed and verified by the authors.
